# Impact of abnormal ambulatory ECG findings when screening for atrial fibrillation in primary care: a qualitative study among participants of the PATCH-AF trial

**DOI:** 10.1136/bmjopen-2025-102160

**Published:** 2025-07-24

**Authors:** Tessa Brik, Marilou S Niekel, Marieke A R Bak, Jelle C L Himmelreich, Ralf E Harskamp, Eric P Moll van Charante

**Affiliations:** 1 Department of General Practice, Amsterdam UMC, University of Amsterdam, Amsterdam, The Netherlands; 2 Department of Ethics, Law and Humanities, Amsterdam UMC, University of Amsterdam, Amsterdam, The Netherlands; 3 Department of Public and Occupational Health, Amsterdam UMC, University of Amsterdam, Amsterdam, The Netherlands

**Keywords:** QUALITATIVE RESEARCH, Cardiovascular Disease, PUBLIC HEALTH, CARDIOLOGY, Primary Health Care, Primary Care

## Abstract

**Abstract:**

**Background and aim:**

European cardiovascular guidelines recommend systematic atrial fibrillation (AF) screening in community-dwelling high-risk patients. However, little is known about the impact of abnormal screening findings, including AF and non-AF incidental findings on the target population. This gap highlights the need to assess both the benefits and potential harms from patients’ perspectives to fully understand the impact of AF screening. Therefore, the aim of this study is to explore patients’ experiences with AF screening among those who received abnormal findings from ambulatory ECG monitoring.

**Design:**

We conducted a qualitative study using semistructured interviews, analysed thematically. Participants in the PATCH-AF trial, based in Amsterdam primary care, were purposively sampled based on their screening results (AF or non-AF incidental findings), sex and socioeconomic status.

**Results:**

We achieved data saturation after conducting 16 interviews (6 with interviewees diagnosed with AF and 10 with non-AF incidental findings). Participants had a median age of 76 (73–79) years, and 56% were male. Their experiences, whether positive or negative, fluctuated throughout the screening process and depended on their initial motivations for participation in AF screening (seeking extra health checks, finding explanations for pre-existing symptoms or contributing to medical research), expectations and perceived benefits from clarification, diagnostic workup or treatment. Influencing factors included the type of finding (AF or non-AF incidental finding), healthcare provider communication and individual characteristics such as age, socioeconomic status and medical history.

**Conclusion:**

This qualitative study highlights both positive and negative AF screening experiences from the patients’ perspective. It underscores how patients’ motivations and expectations for participation, the type of ambulatory ECG finding and communication and follow-up by healthcare providers shape their overall experiences. Healthcare providers should be aware of these factors to optimise screening consultations. Clear guidelines on communicating abnormal ambulatory ECG findings, especially incidental findings, are warranted.

**Trial registeration number:**

The Netherlands Trial Register (NTR) number NL9656.

**STRENGTHS AND LIMITATIONS OF THIS STUDY**

- This study adopts a broad perspective, examining the impact of communication, further diagnostic workup and potential treatment on patients’ experiences with AF screening, within the wider context of sociodemographic differences.
- The selection of interviewees from the ongoing PATCH-AF trial limits the generalisability of the results.
- In our purposive sampling approach, we did not focus on ethnicity as a criterion; nevertheless, it is important to acknowledge that cultural factors can influence patients’ experiences and perspectives.

## Background

 Atrial fibrillation (AF) is the most common cardiac arrhythmia, with prevalence rising from 0.2% at age 45–54 to 17% in those aged 80 and over.^1^ Due to an ageing population, the prevalence of AF is expected to increase accordingly.^2^ AF is associated with stroke, heart failure and cognitive decline, making early detection and effective therapy, including anticoagulation, crucial.^3^ However, diagnosis is challenging due to paroxysmal or asymptomatic episodes. Continuous ambulatory ECG screening helps to detect transient AF that standard ECGs may miss, leading to higher AF detection rates.^4 5^ While European cardiovascular guidelines recommend AF screening in community-dwelling high-risk patients, its impact on stroke reduction remains uncertain.^3 6 7^ Considerations on whether to implement population screening for AF have thus far largely relied on data demonstrating improvement in clinical outcomes through screening. However, to make rational decisions regarding the implementation of such programmes, it is crucial to also consider the potential benefits and harms from patients’ perspective. While studies have explored general practitioners’ (GPs) experiences with AF screening^8^,^12^ and patient reactions to normal AF screening results,^13^ little is known about how patients perceive abnormal screening results. Therefore, this study aims to explore patients’ experiences with receiving abnormal continuous ambulatory ECG findings during AF screening, including AF and non-AF incidental findings.

## Methods

In this qualitative study, we conducted semistructured interviews with a thematic analysis.^14 15^ It is a substudy of the ongoing trial ‘Personalized approach using wearable technology for early detection of atrial fibrillation in high-risk primary care patients (PATCH-AF study)’.^16^ The PATCH-AF trial is a cluster randomised controlled trial, comparing AF screening, with usual care in a high-risk primary care population (65 years or older and a CHA_2_DS_2_-VASc score ≥3 in men and ≥4 in women,^17^ without prior AF, pacemaker or implantable cardioverter-defibrillator) in the Netherlands. The screening intervention consists of 7-day continuous heart rhythm monitoring using the Bittium Faros 360 (a three-lead ambulatory ECG monitoring device, used in accordance with its intended use) conducted annually for three consecutive years. Results of the 7-day heart rhythm monitoring were sent to the participants’ GP and the communication and follow-up of screening results were left to the discretion of the participating general practices, in line with their usual care procedures.

Our qualitative evaluation was informed by a constructivist orientation, assuming that participants construct meaning based on their individual experiences and social context. This perspective aligns with our aim to explore how patients make sense of receiving abnormal ambulatory ECG findings, and guided our thematic analysis of the interviews. This theoretical stance also shaped our open-ended interview approach and iterative theme development (as described in the online supplemental data collection section), with close attention to participants’ own language and interpretations.

### Participants

We included participants from the PATCH-AF trial with screen-positive results, regardless of whether further diagnostics would take place. Screen-positive results included AF or other ambulatory ECG abnormalities (non-AF incidental findings), reported between October 2021 (start of the PATCH-AF study) and December 2023. We excluded patients with severe language barriers, hearing loss or cognitive impairment. Eligible patients were contacted by phone and received an information letter about the study. We conducted interviews with patients diagnosed with AF as well as those with non-AF incidental findings until data saturation was achieved. We applied purposive sampling to identify and select information-rich cases for the most effective use of limited resources.^18^ Selection criteria included sex, social economic status (SES), type of ambulatory ECG finding (AF or non-AF incidental findings) and time between diagnosis and interview, as we hypothesised these factors to significantly influence how individuals reflected on their experiences.^19^

### Data collection

Interviews were conducted between June 2023 and January 2024. A female medical student in her senior year of training (MSN), who had no prior relationship with the participants, and a female GP trainee and PhD student (TB), who had had previous contact with participants as a study investigator in the PATCH-AF trial, conducted the interviews. Both interviewers and interviewees were aware of each other’s professional roles and levels of experience. To ensure consistency in their approach, MSN and TB conducted the first four interviews collaboratively. Interviews were conducted either at the interviewees’ homes or at Amsterdam UMC, depending on the patients’ preferences. The interviews were recorded using a certified Philips Voice Recorder provided by the Amsterdam UMC. At the start of each interview, it was emphasised that we were interested in patients’ experiences, both positive and negative. Each interview started with an open question, followed by further questions tailored to the individual responses. We developed an interview guide using a topic list that encompassed all relevant themes (online supplemental file 1). The topic list was evaluated and adapted when new themes emerged or when new insights prompted follow-up questions. During the interviews, we focused on patients’ motivations to participate, their experiences with receiving ambulatory ECG monitor findings, communication with their healthcare provider, diagnostic tests and treatments, and how these factors influenced their overall experience with AF screening. Interviews were conducted in Dutch. We conducted interviews until we achieved data saturation. This reflects the point where further data collection no longer contributed meaningful new information. After the interview, we sent member checks via email to allow participants to review, correct or adapt their responses. MSN and TB took field notes throughout the research process.

### Coding and analysis

MSN and TB transcribed the interviews verbatim and conducted thematic analysis. Both researchers independently read the transcripts to familiarise themselves with the data, coded the data using the MAXQDA software and generated initial themes. Subsequently, these themes were discussed and reviewed collaboratively until consensus was reached. After this, the research team (TB, MSN, EPMvC) reviewed the themes, after which the interviews were re-read and re-coded where relevant, to identify final themes and ensure that the thematic maps accurately represented the entire dataset. We identified relevant quotations and translated these into English. We reported the results using the Consolidated criteria for Reporting Qualitative research guidelines (online supplemental file 2).^20^ After the analysis was finalised, all audio recordings were deleted.

## Results

We applied an iterative recruitment strategy and invited 24 patients, of whom 6 (25%) declined participation due to lack of interest, time constraints or personal health issues. We recruited participants progressively and conducted interviews with 18 patients from eight different GPs in Amsterdam who participated in the PATCH-AF trial.^16^ We achieved data saturation when interviews did not lead to new perspectives on the existing themes and no new themes emerged. We excluded two of the interviews from our analysis because the interviewees had perceived their results as normal, despite having an abnormal ambulatory ECG monitoring result. The included 16 interviews lasted 19–42 min and interviewees had a median age of 76 (73–79) years (table 1). In 6 interviewees, AF was detected on ambulatory ECG monitoring, while 10 others had incidental non-AF findings, sometimes involving multiple findings, including premature ventricular contractions (n=5), non-sustained ventricular tachycardia (n=4), atrial tachycardia (n=3), second-degree atrioventricular block (n=2), a broad QRS complex (n=1) and premature atrial contractions (n=1).

**Table 1:** Characteristics of interviewees included for analysis (n=16)

Characteristics	Total n=16	AF n=6	Non-AF incidental ECG findings n=10
Sex			
Male	9	4	5
Female	7	2	5
Age median (IQR)	76 (73–79)	72 (67–79)	77 (74–79)
65-75	7	3	4
≥75	9	3	6
Socioeconomic status			
Low	9	3	6
Medium/high	7	3	4
Time between diagnosis and interview			
≤6 months	7	2	5
>6 months	9	4	5
Diagnostic workup			
Yes	9	3	6
No	7	3	4
Treatment			
Yes	5	4	1
No	11	2	9

Semistructured interviews were conducted at the interviewees’ home (n=13) or at Amsterdam UMC (n=3). In some interviews conducted at home, there was a family member or partner of the patient present (n=4).

AF, atrial fibrillation.

**Figure 1:**
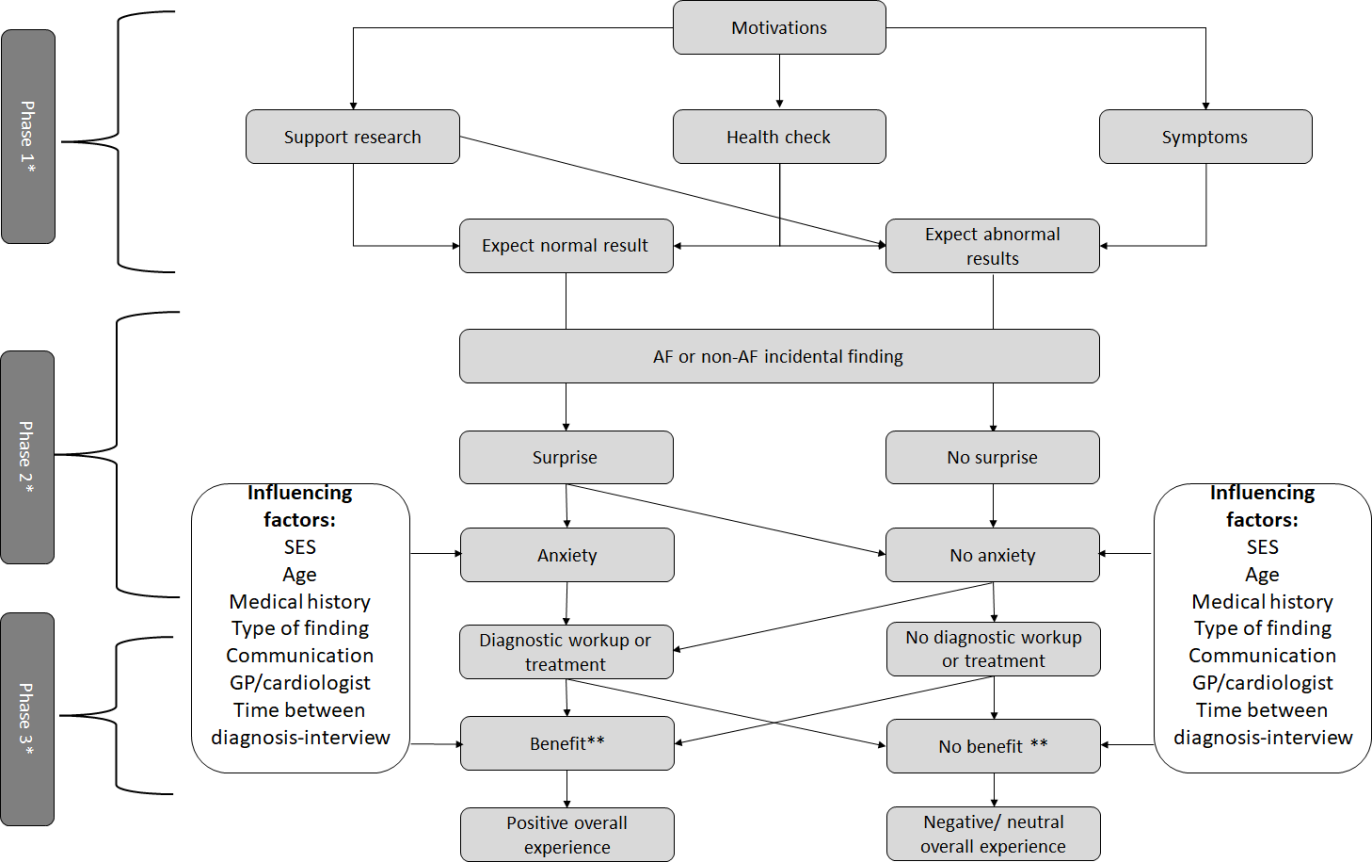
Results. *Phase 1: invitation to and participation in the 7-day heart rhythm screening. Phase 2: receiving the screening results. Phase 3: diagnostic workup and treatment. **Perceived benefit from clarification, diagnostic workup or treatment. AF, Atrial fibrillation; GP, general practitioner; SES, socioeconomic status.

Through these interviews, we identified both positive and negative experiences with AF screening from patients’ perspectives. Experiences fluctuated across different phases of the screening process which could be categorised as follows: invitation to and participation in the 7-day heart rhythm screening, receiving the screening results, and the subsequent diagnostic workup and treatment (figure 1).

### Phase I: invitation to and participation in 7-day heart rhythm monitoring

#### Motivations for participating in AF screening

Upon receiving the invitation for AF screening, interviewees expressed diverse motivations for participation (figure 1). These included appreciating the opportunity for an extra health check, often related to family or medical history, experiencing cardiac symptoms or a desire to contribute to scientific research.

Interviewees who valued the extra health check sought reassurance and considered it important to undergo health checks whenever available.

What I can do to keep my health under control, I will. And then this comes along. (participant 12)

Interviewees with cardiac symptoms expected an explanation for their symptoms.

Well, I hoped for more information about the symptoms, that would be interesting for me. (participant 13)

Individuals who participated out of interest in science had altruistic motives and showed a general interest in the overall findings.

I understand that research, research like this… can be useful and eh…, I am very curious about the results. And I want to contribute to that. (participant 6)

During this first phase, participants motivated by the desire for an extra health check or to contribute to science did not experience any anxiety before or during the 7-day screening itself. However, participants with complaints reported being more aware of symptoms during this period, which sometimes induced anxiety but also sparked curiosity about whether an explanation for these symptoms would be found. In all groups, wearing the heart rhythm device was either experienced neutrally or negatively because of discomfort caused by the plasters or during sleeping.

### Phase II: receiving the screening results

In this phase, we identified different expectations regarding the ambulatory ECG monitor findings. The motivations mentioned previously played a significant role in shaping interviewees’ expectations. Most participants who underwent screening as an extra health check or to contribute to science did not anticipate an abnormal result, as they had not experienced any symptoms. They were often surprised by the diagnosis, which often raised questions and, in some cases, led to distrust in the diagnosis or feelings of anxiety.

No, I really did not expect this… I was very surprised… yes because… I mean, I felt good. I did not notice anything. (participant 15)

Yes, I was really shocked, oh no I thought… (participant 11)

However, interviewees experiencing symptoms expected an abnormal result and perceived the screening as beneficial, as it provided an explanation for their symptoms.

I already suspected it, I feel it, I feel it in my body. (participant 3)

#### Communication by healthcare providers

PATCH-AF participants were instructed to contact their GP for their results after a specified period. However, many interviewees expected their GP to initiate contact if an abnormality had been detected, assuming that no news from the GP implied that no abnormalities had been found.

In most cases, the findings were communicated to interviewees via telephone, primarily by their GPs and occasionally by practice nurses. This phase highlighted the significant impact of tailoring communication to individual preferences on anxiety and reassurance. Some participants sought detailed information for reassurance, while others simply wanted to know whether treatment was necessary. Unclear communication resulted in increased anxiety and concern about their heart condition.

The general practice nurse called on a Friday afternoon to inform me that I had to make an appointment with the GP because of the result. But then I couldn’t get an appointment with the GP that day, so you spend the whole weekend thinking, what did they find? (participant 16)

#### AF versus non-AF incidental findings

Information regarding an AF diagnosis was often clear, but they way GPs communicated this result, led to either expressions of concern about complications such as strokes or to reassurance that AF is not a serious condition.

But, well, there’s a higher risk of stroke now. I don’t think I can prevent that or anything. So, yeah, it’s in my head, I carry that with me, that it can still… well, it can happen to anyone, but the chance is indeed greater. But, yeah, you shouldn’t dwell on that too much because that’s it, then you won’t have a life. (participant 12)

For me it was clear, it was not a very worrisome diagnosis. (participant 14)

Regarding non-AF incidental findings, most interviewees were uncertain whether their ambulatory ECG monitor finding indicated AF or a non-AF finding. Furthermore, it was unclear what clinical implications these findings had and whether diagnostic workup or treatment was needed. This lack of clarity frequently led to confusion and increased worry upon receiving the diagnosis.

Well, I thought, what what what is going on now. (participant 4)

Every now and then it crossed my mind, and I was worrying about whether it is something bad or not. (participant 7)

All interviewees expressed a desire to be informed of any incidental findings, regardless of whether they would be referred for further diagnostic tests or whether the finding was treatable.

Yes, I think it is fine if they tell you that. I don’t have any problems with that. Otherwise, you have a test that is only partially communicated and you still don’t know anything. (participant 2)

Yes, if your GP comes across an incidental finding and chooses not to report it to you… I don’t think that’s the right way to treat patients. In that manner, the GP does not take you seriously. (participant 15)

### Phase III: diagnostic workup and treatment

In the third phase, GPs generally did not conduct additional assessments independently, with only one participant receiving treatment directly from their GP. The remaining participants were either referred to a cardiologist or experienced no further action from their GP regarding the result. This phase highlighted the impact of referral, diagnostic workup and treatment on interviewees’ levels of anxiety and reassurance.

Interviewees referred for further diagnostic workup to the cardiologist expressed a preference for their expertise over that of the GPs’ and found the referral and diagnostic tests reassuring.

Yes, I think I’d trust someone specialised in that a bit more than the GP. (participant 7)

Conversely, most interviewees who were not referred stated that they did not find further referrals or additional diagnostic tests necessary.

Because, very often, when people start digging… it is a fact… then suddenly there are more and more problems. And I think to myself, well… what I don’t know, cannot hurt me. (participant 3)

#### AF vs non-AF incidental findings

As mentioned earlier, interviewees with non-AF incidental findings often found the information provided by their GP about the ambulatory ECG findings unclear, leading to anxiety or uncertainty. However, after undergoing diagnostic workup, they were frequently reassured by the cardiologist that the findings were not a cause for concern.

I was reassured by the cardiologist, he did some tests and told it was nothing to worry about. (participant 1)

Treatment was reassuring for most interviewees with AF because they felt that it reduced the risk of complications and was initiated before the onset of symptoms. They stated that without the screening, such a diagnosis may not have been made.

As I mentioned before, I am very happy I participated and they found something. Of course I am not happy I have a diagnosis, but I am happy they found it, so I can get treatment for it. (participant 10)

Some interviewees diagnosed with AF expressed concerns about treatment side effects, such as the risk of major bleeding.

So then there is the discussion, that the risk of blood clots is decreasing, but the risk of cerebral bleeding is increasing. So, you weigh those risks… but yes, it is what it is… (participant 14)

Interviewees who experienced surprise or anxiety during the second phase were sometimes reassured during the subsequent diagnostic workup or treatment during the third phase, resulting in an overall positive experience. However, for others, anxiety about the prognosis and complications of the disease or treatment persisted—or was even heightened—during the third phase.

Interviewees who participated solely to contribute to science did not receive any treatment and did not experience any personal benefit. Nonetheless, they expressed satisfaction with their contribution to science, reflecting their altruistic motivation for participation.

### Other influencing factors on anxiety, reassurance and coping

In this study, interviewees with low SES, advanced age, an extensive medical history and an extended time between diagnosis and interview tended to be more easily reassured and often coped well with a diagnosis.

I… I wouldn’t know what to worry about. I am at an age where a lot of people have already gone, and then it must have been my time. (participant 8)

It is in your head, but after some time it kind of drifts away and you think, oh well… (participant 16)

Conversely, interviewees with high SES, younger age, a limited medical history and interviews conducted shortly after the diagnosis often tended to experience anxiety and to have difficulty with coping with the diagnosis.

It was not completely normal; something is a little bit off and you never know what will happen. (participant 7)

Well, the fear for a stroke, that eh I did not find that a pleasant experience (participant 14)

Additional quotes of interviewees can be found in online supplemental file 4.

## Discussion

### Main findings

In this qualitative study, interviewees reported both positive and negative experiences with AF screening, which fluctuated during the screening process. Experiences were shaped by their initial motivations, expectations, type of ambulatory ECG findings (AF or non-AF incidental finding), healthcare provider communication and perceived benefits following clarification, advice, referral and/or treatment.

Interviewees who underwent screening for an extra health check or to contribute to medical research were often surprised and sometimes anxious upon receiving abnormal results, as they had no symptoms and had expected reassurance. In contrast, interviewees with pre-existing symptoms anticipated abnormal results and appreciated receiving an explanation for their symptoms.

Interviewees with non-AF incidental findings frequently expressed anxiety mainly due to unclear communication about the implications of their findings. In contrast, those diagnosed with AF generally received a clearer explanation regarding follow-up and clinical implications.

After referral to a cardiologist, those with non-AF findings were generally reassured that their results were not concerning. Among those with AF, some felt reassured by their cardiologist’s explanation that AF is not a worrisome condition, whereas others remained apprehensive about the prognosis. For some, treatment provided reassurance, while others were concerned about potential treatment complications. Several internal factors influenced interviewees’ experiences. Interviewees with low SES, advanced age, extensive medical history and longer interval between diagnosis and interview tended to be easily reassured, cope well with the diagnosis and have an overall positive screening experience.

### Comparison with previous work

#### Motivation and expectation

This study underscores how patients’ motivations to participate in AF screening shape their experiences. Benefitting science is a common motivator for participating in medical research,^21^,^23^ but participants motivated solely by scientific curiosity may not represent broader community attitudes towards integrated screening in clinical care. In contrast, those seeking check-ups or experiencing symptoms likely offer more representative perspectives, mirroring motivations observed in another AF screening trial^24^ and in clinical cancer screening.^25^ Interviewees’ expectations of screening results in our work were influenced by the presence or absence of symptoms, suggesting the need for greater awareness and education about (asymptomatic) AF.^2426^,^29^ Literature demonstrated that patients tend to overestimate the benefits of various screening interventions and are often unaware of possible harms.^2430^,^32^ Practitioners should be mindful of this tendency when discussing screening options with patients and should also mention the possible harms (eg, false positives, induced anxiety, incidental findings with no clear workup protocols) of AF screening.

#### Communication

A recently published qualitative trial studied facilitators and barriers for the implementation of AF screening, and their results underscored the importance of effective communication by the research team.^33^ Furthermore, effective communication is a well-documented determinant of patient satisfaction and healthcare provider-patient relationship. Patients typically rely on specialised expertise and on how diagnoses are communicated, which can significantly impact their experience and level of reassurance.^34 35^ Tailoring communication to individual preferences is crucial; while some participants sought detailed information for reassurance, others simply wanted to know if treatment was necessary.

#### Incidental findings

Interviewees expressed a preference to be informed about any incidental findings, regardless of the need for further referral or treatment. It is thus crucial for GPs to have the expertise to interpret these findings and to provide clear information about their significance, available treatments and potential complications. Currently, there are no clear guidelines on managing incidental findings detected during AF screening, leaving the diagnostic and treatment decisions to the discretion of the GP. The potential for incidental findings, along with the associated risks of overtreatment or anxiety, raises ethical dilemmas regarding the implementation of AF screening. Previous literature on incidental findings during screening has primarily focused on genetic screening.^36^ There, the consensus is that findings can be disclosed, albeit with caution, when clinical utility has been confirmed and when there is an option for prevention or treatment. Moreover, the potential for retrieving incidental findings at all should be limited in advance when implementing a screening programme. If any incidental findings still arise, disclosure policies should help patients make decisions in line with their own values and preferences.^37^ “The Implantable loop recorder detection of atrial fibrillation to prevent stroke (LOOP) Trial’' showed that AF screening with implantable loop recorders led to a sixfold increase in bradycardia diagnoses and a significant increase in pacemaker implantations, but did not find an increased risk of syncope or sudden death. ^38^ These results suggest potential overdiagnosis and overtreatment.

#### Influencing factors

In line with the Health Belief Model, AF screening experiences varied, depending on factors like SES, age, medical history and timing of diagnosis.^19^

### Strengths

This study has several strengths. First, this study adds to the limited evidence on patients’ perspectives on receiving abnormal AF screening results,^33^ and to our knowledge, it is the first study to interview participants with incidental non-AF findings on continuous ambulatory ECG monitoring. Second, we adopted a broad perspective, examining the impact of communication, further diagnostic workup and potential treatment within the wider context of sociodemographic differences.

### Limitations

While the study offers valuable insights into the patients’ experiences in AF screening, several limitations need to be acknowledged. First, the interviewees were selected from the ongoing PATCH-AF trial, potentially introducing selection bias. Those expressing interest in both the trial and the interview study may have had a more positive view of AF screening or a stronger affinity for medical research. Second, although we included patients from diverse general practices across different neighbourhoods in Amsterdam, the findings may not sufficiently reflect those from more urban settings. Third, in our purposive sampling approach, we did not focus on ethnicity as a criterion to avoid introducing potential unknown, cultural heterogeneity in expectations and preferences.^39^ Nevertheless, it is important to acknowledge that cultural and regional factors can influence patients’ experiences and perspectives on healthcare, warranting future research to validate our findings in other subgroups. Fourth, this study relied on interviewees’ recollections, potentially introducing recall bias. We included interviewees who received their screening findings between 3 weeks and 18 months ago, as we expected time to influence their experiences. This induced heterogeneity in the results but also gave us the opportunity to study both short-term and long-term effects of AF screening, which strengthens our results. Fifth, the number of interviews with participants with incidental findings was larger than that with participants with AF, as the former group required a larger sample to reach data saturation—likely due to heterogeneity in incidental findings. At last, it is important to note that, with this qualitative study, we aimed to observe patterns and contrasts rather than establish associations.

### Implications for clinical practice and future research

#### Clinical practice

Our findings underscore the importance for healthcare providers and researchers in screening trials to explore patients’ motivations and expectations before initiating rhythm monitoring or AF screening. This allows them to assess whether testing aligns with patients’ perceptions and to tailor communication of abnormal results—including asymptomatic AF or non-AF incidental findings—to the individual’s context and information needs. Education on the possible consequences of such tests, including uncertainty or detection of unexpected findings, could improve patient understanding. Moreover, greater awareness and knowledge of incidental findings among healthcare providers could mitigate unnecessary patient concerns.

#### Future research

Consistency in reporting and follow-up of incidental findings in AF screening trials will help establish evidence-based recommendations for future clinical guidelines. If future clinical guidelines are to recommend AF screening and reporting incidental findings, the development of a decision aid tool—similar to other screening strategies in healthcare—will be essential for patients considering AF screening.

Future qualitative studies could focus on AF screening participants who experience symptom relief through treatment, as well as those who encounter major adverse events, to explore how such outcomes shape their views on screening.

Furthermore, the main ethical question remains whether the potential harms of AF screening outweigh its expected benefits in survival and quality of life, warranting further research.^40^

#### Conclusion

This qualitative study highlights that interviewees who participated in AF screening had both positive and negative experiences, which fluctuated during the different phases of the screening process. It underscores how patients’ motivations and expectations for participation, the type of ambulatory ECG finding, healthcare provider communication and perceived benefit following clarification, diagnostic workup or treatment shape their overall experiences. Healthcare providers should be aware of these factors to optimise screening consultations and to tailor communication to individual needs when discussing abnormal ambulatory ECG findings, especially non-AF incidental findings. Clear guidelines on this communication are warranted. Future research should further explore the potential downsides of screening, as well as the implications of diagnostic workup and/or treatment following incidental findings.

## Supplementary material

10.1136/bmjopen-2025-102160 online supplemental file 1

10.1136/bmjopen-2025-102160 online supplemental file 2

10.1136/bmjopen-2025-102160 online supplemental file 3

## Data Availability

Data are available upon reasonable request.
