# Supplementary material for: Impact of abnormal ambulatory ECG findings when screening for atrial fibrillation in primary care: a qualitative study among participants of the PATCH-AF trial
Source: BMJ Open. 2025 Jul 24;15(7):e102160. doi: 10.1136/bmjopen-2025-102160 (PMC12306208; doi:10.1136/bmjopen-2025-102160)
Supplement: online supplemental file 1 [file bmjopen-15-7-s001.docx]

**Supplement 1. Interview/topic guide.**

**Introduction:**

Researchers introduce themselves and explain their professional roles. Researchers ask the participant if the received information about the study was clear and ask if the participant has any questions.

Researchers obtain informed consent prior to the interview. Researchers explain that they are interested in the participants own experience whether positive or negative.

**Topics**

*Overall experience*

- Can you tell me about your experiences with the PATCH-AF study?

- Positive/ negative aspects?

- Surprising/ challenging aspects?

*Motivation*

- What motivated you to participate in the study?
- Any concerns or hesitations?

*Expectation*

- What expectations did you have about the screening?
- Why did you have those expectations?
- Expected benefits?
- Expected harms?

*Holter monitoring*

- How would you describe your experience with the Holter monitoring?
- Physically
- Emotionally

*Receiving results*

- Can you describe how you received the results of the study?
- Can you tell me about your conversation with your general practitioner or assistant regarding the results?
- Clear information about the findings?
- How important were the specific details of the findings to you?
- How did you perceive the seriousness of the condition and its consequences?
- How did you feel after receiving the results?
- Relief/ reassurance/ concerns /anxiety?
- What contributed to these feelings?
- Perceived benefits? Perceived harms?
- When you participated in the screening for AF, how aware were you that we might detect other rhythm abnormalities?
- How do you feel about receiving an unexpected finding?
- In your opinion, should healthcare providers discuss incidental findings with participants?

*Referral*

- Has your GP referred you to a cardiologist?
- Can you tell me about this experience?
- How did the referral process unfold, and how did it affect you?
- If the GP did not refer you, how did that affect you?

*Diagnostic workup and treatment*

- How would you describe your experience with the follow-up tests and any treatments you received?
- Benefits/ side effects/ treatment concerns

*Influence of screening results on participant*

- How are you feeling now, some time after participating in the study?
- Overall experience different now compared to when you received the results?
- In what ways has participating in the study, receiving the results, and undergoing treatment affected how you view your health?
- Anything else you like to share?

**Interview close**

Researchers thank participants for their participation and ask participants if they have any questions.

Researchers ask if the participant like to receive a member check.

Researchers leave contact details for any questions or comments.
