# Supplementary material for: Impact of abnormal ambulatory ECG findings when screening for atrial fibrillation in primary care: a qualitative study among participants of the PATCH-AF trial
Source: BMJ Open. 2025 Jul 24;15(7):e102160. doi: 10.1136/bmjopen-2025-102160 (PMC12306208; doi:10.1136/bmjopen-2025-102160)
Supplement: online supplemental file 3 [file bmjopen-15-7-s003.docx]

| **Main themes** | **Subthemes** | **Quotes** |
| --- | --- | --- |
| Phase 1: Invitation to- and participation in the seven-day heart rhythm screening | -Motivations | "You can bury your head in the sand, or you can simply say 'yes' when the opportunity presents itself." (Participant 4)  **“Well, I hoped for more information about the symptoms, that would be interesting for me” (Participant 13)**  “Well… because in my family a lot of women have heart problems and passed away. So I thought well now I can find out whether I also have heart problems.” (Participant 11)  “Like I mentioned before, research in general is of course very important. And eh, medical research.. well if I can contribute in more knowledge, than I am happy to participate. So that is my motivation basically.” (Participant 16)  “The reason to participate is that I have high blood pressure and diabetes, so I thought it is a welcome plus to participate in this study.”(Participant 12)  **“What I can do to keep my health under control, I will. And then this comes along.” (Participant 12)**  **“I understand that research, research like this… can be useful and eh..., I am very curious about the results. And I want to contribute to that. “(Participant 6)** |
| Phase 2: Receiving the screening results | -Expectations  -Communication by health care provider  -Type of ambulatory ECG finding | “Eh, I did not expect this result, because I don’t experience any symptoms” (Participant 14)  **“Yes, I was really shocked, oh no I thought…” (Participant 11)**  “Something like… everything will be fine, you know. I participate to this study but eh, I expect everything to be fine.” (Participant 10)  **“No, I really did not expect this… I was very surprised... yes because... I mean, I felt good. I did not notice anything” (Participant 15)**  **“I already suspected it, I feel it, I feel it in my body” – (Participant 3)**  "So the result is just: ‘something was found.’ But it doesn’t say where, how, or when. So as a patient, I’m left guessing." (Participant 13)  “We discussed with my GP that certain diagnostic test will lead to another considerable amount of test with consequences. And I don’t want that anymore.”  (Participant 6)  “ I really had to ask the GP about the results you sent to him.. He is probably busy and that is a societal problem, but it was inconvenient for me.” (Participant 13)  “So I experienced a bit of a panic attack, well “panic attack”, but eh, everything went very fast. The GP that called and said that I needed to go to the cardiologist. And the cardiologist called right away. Now I think they probably didn’t have any patients at that moment and thought yay there we go...”  (Participant 4)  **"The general practice nurse called on a Friday afternoon to inform me that I had to make an appointment with the GP because of the result. But then I couldn't get an appointment with the GP that day, so you spend the whole weekend thinking, what did they find?" (Participant 16)**  **“Well, I thought, what what what is going on now” (participant 4)**  **“For me it was clear, it was not a very worrisome diagnosis.” (Participant 14)**  “If they see something else (other than AF), I would also like to know that, yes” (Participant 1)  **“Every now and then it crossed my mind, and I was worrying about whether it is something bad or not.” (participant 7)**  **“Yes, I think it is fine if they tell you that. I don’t have any problems with that. Otherwise, you have a test that is only partially communicated and you still don’t know anything” – (Participant 2)**  **“Yes, if your GP comes across an incidental finding and chooses not to report it to you... I don’t think that’s the right way to treat patients. In that manner, the GP does not take you seriously.” (Participant 15)** |
| Phase 3 : Diagnostic workup and treatment | -Referral  -Experienced benefit/downsides  Coping / anxiety | “Hm, it (referral to cardiologist) was kind of useless… He (cardiologist) only gets the information that I have “fibrillation” and he has a standard treatment for it, so he prescribed that. He did some check-ups, but I don’t even remember what. It didn’t really amount to much." (Participant 14)  **"Yes, I think I'd trust someone specialized in that a bit more than the GP." – (Participant 7)**  **“I was reassured by the cardiologist, he did some tests and told it was nothing to worry about.” (Participant 1)**  **“Because, very often, when people start digging... it is a fact... then suddenly there are more and more problems. And I think to myself, well... what I don’t know, cannot hurt me.” – (Participant 3)**  "The downside is that the blood thinners that I use now, cost a fortune. (participants 14)  “Well, I think it is great they found it. I hope to become 120 years old and healthy.” (Participant 9)  **“As I mentioned before, I am very happy I participated and they found something. Of course I am not happy I have a diagnosis, but I am happy they found it, so I can get treatment for it.” (Participant 10)**  "Well… you're getting older. You just accept more easily that new problems occur” (Participant 5)  "I often find myself wondering: how healthy am I really?” (Participant 12)  “I just take one more medication and I am fine with it.” (Participant 9)  **“So then there is the discussion, that the risk of blood clots is decreasing, but the risk of cerebral bleeding is increasing. So, you weigh those risks... but yes, it is what it is...” (Participant 14)**  **"I... I wouldn't know what to worry about. I am at an age where a lot of people have already gone, and then it must have been my time – (Participant 8)**  **“It is in your head, but after some time it kind of drifts away and you think, oh well” ... (Participant 16)**  **“It was not completely normal; something is a little bit off and you never know what will happen.” (participant 7)**  **“Well the fear for a stroke, that eh I did not find that a pleasant experience” (participant 14)**  **"But, well, there's a higher risk of stroke now. I don't think I can prevent that or anything. So, yeah, it's in my head, I carry that with me, that it can still... well, it can happen to anyone, but the chance is indeed greater. But, yeah, you shouldn't dwell on that too much because that's it, then you won't have a life." (Participant 12)** |
