# Supplementary material for: Impact of abnormal ambulatory ECG findings when screening for atrial fibrillation in primary care: a qualitative study among participants of the PATCH-AF trial
Source: BMJ Open. 2025 Jul 24;15(7):e102160. doi: 10.1136/bmjopen-2025-102160 (PMC12306208; doi:10.1136/bmjopen-2025-102160)
Supplement: Supplementary file 4 [file bmjopen-2025-102160.reviewer_comments.pdf]

PEER REVIEW HISTORY

BMJ Open publishes all reviews undertaken for accepted manuscripts. Reviewers are asked to complete a checklist review form and are provided with free text boxes to elaborate on their assessment. These free text comments are reproduced below.

ARTICLE DETAILS

Title (Provisional)

The impact of abnormal ambulatory ECG findings when screening for atrial fibrillation in primary care: a qualitative study among participants of the PATCH-AF trial

Authors

Brik, Tessa; Niekel, Marilou S.; Bak, Marieke A.R.; Himmelreich, Jelle C.L.; Harskamp, Ralf; Moll van Charante, Eric P

VERSION 1 - REVIEW

**Reviewer**	**1**
**Name**	**Callanan, Aileen**
**Affiliation**	**University College Cork, School of Public Health**
**Date**	**04-Apr-2025**
**COI**	**None**

This is a very interesting and timely qualitative research study.

A little bit more information on the device in the background or methods would be helpful. Mostly to clarify that it is a 7 day continuous holter device as it is firstly refered to as a ECG then a holter. Its a bit unclear if the result is a screen positive result only, or if further diagnostic testing was conducted and confirmed. Is the population that were interviewed all screen positive results only or screen positive results with a further confirmed diagnosis or both? This could also potentially be an influencing factor as identified by you. Further information on the performance of the device in the background would also strengthen the argument for its use as an intervention.

How did you decide that data saturation had been achieved? A bit more context to this would make it clearer.

What language were the interviews conducted in? When exactly were the recordings deleted, after transcription, after analysis?

A table or grid giving an overview of the identified themes and subthemes would also enhance the results section.

The discussion could include some reference to the Wilson and Jungner criteria for screening as this study addresses some key points such as acceptability to patients and risks versus benefits which is mentioned in the future research question.

Reviewer 2

Name Wong, Kam Cheong

Affiliation The University of Sydney Faculty of Medicine and Health, Westmead Applied Research Centre

Date 31-May-2025

COI None

---

1. The authors argued that the participants' experiences were shaped by their initial motivations, etc., which are substantiated by their findings. However, this argument could be strengthened if the authors had included and explained their theoretical orientation that underpins the qualitative evaluation, e.g. constructivism or critical realism. Please note that specifying a theoretical orientation in performing the qualitative assessment is one of the requirements in the COREQ checklist.

2. The authors claim that "This is the first study to conduct interviews with AF screening participants who had abnormal screening results, including AF and non-AF incidental findings." This statement and the corresponding sentences in the Background section may need to be revised, knowing that there were similar publications in the literature, including BMJ Open (https://bmjopen.bmj.com/content/14/10/e088260) and PhD theses (such as Modi RN. Delivering a screening programme for atrial fibrillation: a mixed methods investigation. University of Cambridge, 2023). The literature has included interviews with patients who received abnormal findings, such as atrial fibrillation (AF). (https://ses.library.usyd.edu.au/handle/2123/33258).

3. Under the section "Other influencing factors on anxiety, reassurance and coping", the authors contrasted the different reactions or coping mechanisms between participants from low and high socioeconomic status (SES). The authors included quotes from low SES but did not include quotes from high SES participants. It would be more informative if they could include both.

4. The authors report that participants' expectations (depending on their medical condition and preexisting morbidities) shaped their experience with the program. How can this finding be applied to clinical practice or a future program (e.g.. education or

support provided to participants, in addition to what is explained under the section "Implications for clinical practice and future program")?

5. The authors elaborated the importance of effective communication in conveying findings and educating participants about the findings. Communication was also highlighted as a key determinant in a centrally coordinated and supported screening program reported in BMJ Open (https://bmjopen.bmj.com/content/14/10/e088260). The authors could compare their findings in the discussion.

6. What was the length of the interviews (minutes)? Can this be included in the report?

7. The selected quotes were limited (partly due to the space in the main text). Can participants' quotes be included as supplemental materials?

8. Please include a completed COREQ checklist.

Reviewer **3**

Name **Jones, Nicholas**

Affiliation **University of Oxford, Primary Care Health Sciences**

Date **02-Jun-2025**

COI **I am part of a team conducting a randomised controlled trial of screening among people with atrial fibrillation.**

Thank you for the opportunity to read this work. I think you've identified an important topic given the interest in screening for AF and the lack of research into how the findings are viewed by those who are screened.

Major

- The abstract and methods suggests data saturation was achieved after 16 interviews. This seems at odds with the start of the results, which reports 24 patients were invited, of whom 18 agreed to participate with two subsequently excluded. It seems fortuitous if the 16 people who agreed to take part also were the right number for data saturation? It also seems hard to be sure data saturation was achieved eg. among the six people who had AF diagnosed? Could you comment on the success of your sampling approach given the proportion of people you include with AF v non-AF - was that mix intentional?

- Leading on from this - numbers within sub-groups are very small - can we draw conclusions about factors such as socioeconomic status and how people interpret their AF result among the 6 people this applies to? I found the last statement in the results (starting 'Conversely high SES, younger age...') seemed to gloss over a number of potentially important factors with no supporting quotes or information.

- The study aim is to explore patients' experiences with receiving abnormal screening results, yet it is difficult to know how participants had the information delivered to them. For example - some people were told by the practice nurse but it's not clear if the skill level of the nurse or the format of that discussion. The heterogeneity of how results were fed back makes it hard to know how important other factors truly were, such as the participants initial motivation to join the study.

- It is unclear the importance of some of the non-AF incidental findings from the description given, which may have impacted upon how patients' perceived the result. For example 2nd degree AV block would obviously have different implications if it were type 1 or 2, whilst the duration of non-sustained VT would also be crucial to treatment. Could you report if any patients needed treatment for these findings and whether they felt differently?

- Did patients comment on what treatment they received for AF and whether this improved their symptoms (amongst those this was relevant)? One might think that group would be the most positive about screening if it actually led to them being diagnosed and feeling better. Presumably no patients reported a major adverse event eg bleeding after receiving an anticoagulant?

- The implications for practice suggest clinicians need to provide more comprehensive information to patients but an alternative conclusion would be that the screening study should provide more robust and detailed information that is consistent between participants.

Minor

- I think you should add that only 6 of your participants were found to have AF in the abstract.

- I would be explicit that you will refer to the Bittium Faros 360 as a Holter throughout the rest of the paper

- I would add some references supporting your thematic analysis approach and why this was felt to be the most appropriate method.

VERSION 1 - AUTHOR RESPONSE

Reviewer: 1
Mrs. Aileen Callanan, University College Cork
Comments to the Author:

Comment 1.1 This is a very interesting and timely qualitative research study.
A little bit more information on the device in the background or methods would be helpful. Mostly to clarify that it is a 7 day continuous holter device as it is firstly refered to as a ECG then a holter.

Answer 1.1
Thank you very much for taking the time to read and revise our manuscript and thankyou for your valuable feedback.

We provide additional information on the device in the method section and added the following sentence *"The screening intervention consists of seven-day continuous heart rhythm monitoring using the Bittium Faros 360™ (a three-lead ambulatory ECG monitoring device, used in accordance with its intended use) conducted annually for three consecutive years." (Page 3).* Furthermore, we changed the term Holter and ECG into *'ambulatory ECG monitoring'* and made this consistent throughout the paper.

Comment 1.2 Its a bit unclear if the result is a screen positive result only, or if further diagnostic testing was conducted and confirmed. Is the population that were interviewed all screen positive results only or screen positive results with a further confirmed diagnosis or both? This could also potentially be an influencing factor as identified by you.

Answer 1.2
The population consisted of participants with screen-positive results only, regardless of whether further diagnostics would take place. We added the sentence *"We included participants from the PATCH-AF trial with screen-positive results, regardless of whether further diagnostics would take place. Screen positive results included AF or other ambulatory ECG abnormalities (non-AF incidental findings), reported between October 2021 (start of the PATCH-AF study) and December 2023."* (Page 4).

Comment 1.3 Further information on the performance of the device in the background would also strengthen the argument for its use as an intervention.

Answer 1.3 The device is used to detect AF in usual primary care in the Netherlands. In the PATCH-AF study, the device it is used in accordance with its intended use. *We added that in the following sentence. "…Bittium Faros 360™ (a three lead ambulatory ECG monitoring device, used in accordance with its intended use)…"*(Page 3). Additional information on the device could also be retrieved in the study protocol of the main PATCH-AF trial (Reference 14).

Comment 1.4 How did you decide that data saturation had been achieved? A bit more context to this would make it clearer.

Answer 1.4 We added the following sentences in the Methods and Results sections respectively: *"We conducted interviews until we achieved data saturation. This reflects the point in the analysis where further data collection no longer contributed meaningful new information"*(Methods, Page 4) and
"We achieved data saturation when interviews did not lead to new perspectives on the existing themes and no new themes emerged." (Results, Page 5).

Comment 1.5 What language were the interviews conducted in?

Answer 1.5 Interviews were conducted in Dutch. We added the following sentence *"Interviews were conducted in Dutch."* (Page 4).

Comment 1.6 When exactly were the recordings deleted, after transcription, after analysis?

Answer 1.6 Audio recordings were deleted straight after the analysis. We changed the existing text into *"after the analysis was finalized, all audio recordings were deleted."* (Page 5).

Comment 1.7 A table or grid giving an overview of the identified themes and subthemes would also enhance the results section.

Answer 1.7
Thank you for this suggestion. We had submitted a figure illustrating the thematic structure (Figure 1), but we now realize this may not have been sufficiently clear as a structured overview. We have therefore added a supplementary table (Supplement 4) presenting the main themes and subthemes in a concise grid format, which complements the existing figure and enhances clarity. In response to the comments of the other reviewers, we also included additional quotes in the table.

Comment 1.8 The discussion could include some reference to the Wilson and Jungner criteria for screening as this study addresses some key points such as acceptability to patients and risks versus benefits which is mentioned in the future research question.

Answer 1.8 We added the reference at the sentence "*Furthermore, the main ethical question remains whether the potential harms of AF screening outweigh its expected benefits in survival and quality of life, warranting further research". (1)* (Page 15).

Reviewer: 2
Dr. Kam Cheong Wong, The University of Sydney Faculty of Medicine and Health, The University of Sydney Faculty of Medicine and Health
Comments to the Author:

Comment 2.1 The authors argued that the participants' experiences were shaped by their initial motivations, etc., which are substantiated by their findings. However, this argument could be strengthened if the authors had included and explained their theoretical orientation that underpins the qualitative evaluation, e.g. constructivism or critical realism. Please note that specifying a theoretical orientation in performing the qualitative assessment is one of the requirements in the COREQ checklist.

Answer 2.1
Thank you very much for taking the time to read and revise our manuscript and thank you for your this valuable comment. We agree that specifying our theoretical orientation adds clarity to the interpretative framework of the study. In line with the COREQ recommendations, we have now included this in the manuscript (Methods, p. 3).

“Our qualitative evaluation was informed by a constructivist orientation, assuming that participants construct meaning based on their individual experiences and social context. This perspective aligns with our aim to explore how patients make sense of receiving abnormal ECG findings, and guided our thematic analysis of the interviews.
This theoretical stance also shaped our open-ended interview approach and iterative theme development (as described in the methods section), with close attention to participants’ own language and interpretations.” (Page 3).

Comment 2.2 The authors claim that “This is the first study to conduct interviews with AF screening participants who had abnormal screening results, including AF and non-AF incidental findings.” This statement and the corresponding sentences in the Background section may need to be revised, knowing that there were similar publications in the literature, including BMJ Open (https://bmjopen.bmj.com/content/14/10/e088260) and PhD theses (such as Modi RN. Delivering a screening programme for atrial fibrillation: a mixed methods investigation. University of Cambridge, 2023). The literature has included interviews with patients who received abnormal findings, such as atrial fibrillation (AF). (https://ses.library.usyd.edu.au/handle/2123/33258).

Answer 2.2
Thank you for pointing out this literature. We removed the sentence from page one, we changed the sentence at page 13 and added the citation. *“.. this study adds to the limited evidence on patients’ perspectives on receiving abnormal screening results (2) and to our knowledge, it is the first study to interview participants with incidental non-AF findings on continuous ambulatory ECG monitoring.” (Page 13)*

Comment 2.3 Under the section “Other influencing factors on anxiety, reassurance and coping”, the authors contrasted the different reactions or coping mechanisms between participants from low and high socioeconomic status (SES). The authors included quotes from low SES but did not include quotes from high SES participants. It would be more informative if they could include both.

Answer 2.3
We added the following two quotes at page 11.
“It was not completely normal; something is a little bit off and you never know what will happen.” (participant 7)
“Well, the fear for a stroke, that eh, I do not find that a pleasant thought” (participant 14)

comment 2.4 The authors report that participants’ expectations (depending on their medical condition and preexisting morbidities) shaped their experience with the program. How can this finding be applied to clinical practice or a future program (e.g.. education or support provided to participants, in addition to what is explained under the section “Implications for clinical practice and future program”)?

Answer 2.4

In our revised implications for clinical practice and future research, we clarified how patients' expectations can inform both screening programs and routine care. We now also emphasize their relevance beyond formal AF screening trials, for instance when patients undergo opportunistic rhythm monitoring in general practice or ambulatory settings (e.g. Holter monitoring, wearable devices). We changed the first part into:

"Our findings underscore the importance for healthcare providers and researchers in screening studies to explore patients' motivations and expectations before initiating rhythm monitoring or AF screening. This allows them to assess whether testing aligns with patients' perceptions and to tailor communication of abnormal results—including asymptomatic AF or non-AF incidental findings—to the individual's context and information needs. Education on the possible consequences of such tests, including inducing uncertainty or detection of unexpected findings, could improve patient understanding." (page 14)

Comment 2.5 The authors elaborated the importance of effective communication in conveying findings and educating participants about the findings. Communication was also highlighted as a key determinant in a centrally coordinated and supported screening program reported in BMJ Open (https://bmjopen.bmj.com/content/14/10/e088260). The authors could compare their findings in the discussion.

Answer 2.5
Thank you for this suggestion, we included the following sentence and reference: " *A recently published qualitative trial, studied facilitators and barriers for the implementation of AF screening and, their results underscored the importance of effective communication by the research team. (2)" (page 12)*

Comment 2.6. What was the length of the interviews (minutes)? Can this be included in the report?

Answer 2.6 *"The included 16 interviews lasted 19-42 minutes and…"*(Page 5).

Comment 2.7. The selected quotes were limited (partly due to the space in the main text). Can participants' quotes be included as supplemental materials?

Answer 2.7 We added additional quotes as supplemental material (supplement 4)

Comment 2.8. Please include a completed COREQ checklist.

Answer 2.8 COREQ checklist is included (supplement 2)

Reviewer: 3
Dr. Nicholas Jones, University of Oxford
Comments to the Author:
Thank you for the opportunity to read this work. I think you've identified an important topic given the interest in screening for AF and the lack of research into how the findings are viewed by those who are screened.

Major

Comment 3.1 -The abstract and methods suggests data saturation was achieved after 16 interviews. This seems at odds with the start of the results, which reports 24 patients were invited, of whom 18 agreed to participate with two subsequently excluded. It seems fortuitous if the 16 people who agreed to take part also were the right number for data saturation? It also seems hard to be sure data saturation was achieved eg. among the six people who had AF diagnosed? Could you comment on the success of your sampling approach given the proportion of people you include with AF v non-AF - was that mix intentional?

Answer 3.1

Thank you very much for taking the time to read and revise our manuscript and thank you for your valuable feedback and this thoughtful comment. We agree that clarity about the sampling process and data saturation is essential. In this qualitative study, we applied an iterative recruitment strategy, consistent with standard practice in thematic analysis. Rather than determining a fixed sample size in advance, we recruited participants progressively and continued interviewing until we reached data saturation—the point at which no new codes or themes emerged, and existing themes were well-developed. We initially approached 24 individuals, of whom 18 consented to participate. Two interviews were excluded after completion due to insufficiently meeting inclusion criteria. After completing 16 **eligible** interviews, we determined that data saturation had been achieved, based on careful assessment of the richness and recurrence of themes during analysis. From that point, further recruitment was not required.

We made some adjustments in the result section to make this clearer in the manuscript.

"We applied an iterative recruitment strategy and invited 24 patients, of whom six (25%) declined participation due to lack of interest, time constraints, or personal health issues. We recruited participants progressively and conducted interviews with 18 patients from eight different general practitioners (GPs) in Amsterdam who participated in the PATCH-AF trial (3). We achieved data saturation when interviews did not lead to new perspectives on the existing themes and no new themes emerged." (page 5)

With regard to the mix of participants with AF versus non-AF findings: this distribution was not entirely random. We used purposive sampling to ensure variation in diagnostic outcomes (AF and non-AF), as we expected this factor to influence patient experiences. While the group with AF was smaller (n=6), thematic saturation within this subgroup was supported by the recurrence and consistency of key experiences across participants. Possibly, the relative homogeneity of experiences among participants diagnosed with AF contributed to earlier thematic saturation within this subgroup. In contrast, the greater variability in types of incidental findings among non-AF participants may have led to a wider range of perspectives, thereby requiring a larger number of interviews to reach data saturation. We acknowledge the smaller number of interviews conducted with participants diagnosed with AF and have now added a sentence in the discussion to reflect this as a limitation and to guide interpretation of our findings:

"The number of interviews with participants with incidental findings was larger than with participants with AF, as the former group required a larger sample to reach data saturation -likely due to heterogeneity in incidental findings." (Page 14)

Comment 3.2 Leading on from this - numbers within sub-groups are very small - can we draw conclusions about factors such as socioeconomic status and how people interpret their AF result among the 6 people this applies to? I found the last statement in the results (starting 'Conversely high SES, younger age...') seemed to gloss over a number of potentially important factors with no supporting quotes or information.

Answer 3.2

Thank you for this important observation. We fully acknowledge that the number of participants in each subgroup—particularly those diagnosed with AF—is small, and we do not intend to make generalisable claims based on these findings. Rather, our aim was to explore patterns and contrasts in how participants made sense of their experiences, and how factors such as socioeconomic background and age might shape these interpretations.
The statement at the end of the results ("Conversely, high SES, younger age...") was intended to tentatively reflect a pattern observed across several interviews, but we agree it could be seen as overstated without direct support. We have therefore revised this passage to clarify that this was a suggestive observation, not a conclusion, and have now added supporting quotes in the manuscript as well as supplementary data to illustrate the perspective described.

"Conversely interviewees with high SES, younger age, a limited medical history and interviews conducted shortly after the diagnosis often tended to experience anxiety and to have difficulty with coping with the diagnosis." (Page 11)

Added quotes in the manuscript:

"It was not completely normal; something is a little bit off and you never know what will happen." (participant 7)

"Well the fear for a stroke, that eh, I do not find that a pleasant experience" (participant 14)

(Page 11)

We also note this limitation in the discussion.
"At last, it is important to note that, with this qualitative study, we aimed to observe patterns and contrasts rather than establish associations." (page 14)
This approach aligns with the exploratory nature of qualitative research, where such reflections can help generate hypotheses for future studies with larger samples.

Comment 3.3 The study aim is to explore patients' experiences with receiving abnormal screening results, yet it is difficult to know how participants had the information delivered to them. For example - some people were told by the practice nurse but it's not clear if the skill level of the nurse or the format of that discussion. The heterogeneity of how results were fed back makes it hard to know how important other factors truly were, such as the participants initial motivation to join the study.

Answer 3.3
Thank you for this insightful comment. The PATCH-AF trial was designed to reflect routine primary care practice as closely as possible. As such, the follow-up of screening results—including who communicated the findings and how this was done—was left to the discretion of the participating general practices, in line with their usual care procedures. This inevitably introduced some variation in how information was delivered.

We agree that the way in which results are communicated—whether by a GP or a practice nurse—can shape how participants experience the screening process. While we did not collect objective data on the communication style, skill level, or content of these consultations, this was beyond the scope of the current study. Our qualitative analysis focuses on patients' subjective experiences, which are valuable precisely because they capture how communication was perceived by

participants, regardless of professional background or communication training of the healthcare provider.
We acknowledge the heterogeneity in result delivery and have clarified communication process in the manuscript. Rather than viewing it as a limitation, we believe it reflects real-world complexity and helps illuminate how different factors—such as initial motivation and the manner of feedback—interact to shape patients' experiences over the course of the screening process.

"Results of the seven-day heart rhythm monitoring were sent to the participants' GP and the communication and follow-up of screening results was left to the discretion of the participating general practices, in line with their usual care procedures." (Page 3)

Comment 3.4 It is unclear the importance of some of the non-AF incidental findings from the description given, which may have impacted upon how patients' perceived the result. For example 2nd degree AV block would obviously have different implications if it were type 1 or 2, whilst the duration of non-sustained VT would also be crucial to treatment. Could you report if any patients needed treatment for these findings and whether they felt differently?

Answer 3.4
We have added information to Table 1 (page 17) indicating whether patients received further diagnostic work-up and or treatment following their incidental findings. We agree that the clinical significance and severity of a finding -such as the distinction between type I and type II second-degree AV block- can influence how patients perceive their result. However, given the heterogeneity and small number of non-AF incidental findings in our sample, it was beyond the scope of this study to systematically compare perception by specific subtype. Our primary focus was on the shared experiences and general implications of receiving an unexpected result, rather than on clinical stratification.

Comment 3.5 Did patients comment on what treatment they received for AF and whether this improved their symptoms (amongst those this was relevant)? One might think that group would be the most positive about screening if it actually led to them being diagnosed and feeling better. Presumably no patients reported a major adverse event eg bleeding after receiving an anticoagulant?

Answer 3.5
No patients indicated that they experienced any physical benefit from the medication, but some indicated to benefit from early diagnosis in terms of prevention of complications. We also observed that participants with symptoms and screen positive result were generally positive about screening because their results matched their expectation. No patients reported major adverse events after receiving an anticoagulant. We added the following sentence in the discussion.
"Future qualitative studies could focus on AF screening participants who experience symptom relief through treatment, as well as those who encounter major adverse events, to explore how such outcomes shape their views on screening."(page 15).

Comment 3.6 The implications for practice suggest clinicians need to provide more comprehensive information to patients but an alternative conclusion would be that the screening study should provide more robust and detailed information that is consistent between participants.

Answer 3.6
We agree with this alternative conclusion and added this in the following sentences:

“Our findings underscore the importance for healthcare providers and researchers in screening studies to explore patients' motivations and expectations before initiating rhythm monitoring or AF screening. This allows them to assess whether testing aligns with patients’ perceptions and to tailor communication of abnormal results—including asymptomatic AF or non-AF incidental findings—to the individual’s context and information needs.” (Page 14)

Minor

Comment 3.7 I think you should add that only 6 of your participants were found to have AF in the abstract.

Answer 3.7

We added this in the abstract: *“We achieved data saturation after conducting 16 interviews (6 interviewees with AF and 10 with non-AF incidental findings).”(*page 1)

Comment 3.8 I would be explicit that you will refer to the Bittium Faros 360 as a Holter throughout the rest of the paper

Answer 3.8 Taking in account the comment of reviewer one, we changed the term Holter into ‘ambulatory ECG monitoring’ and made this consistent throughout the paper.

Comment 3.9 I would add some references supporting your thematic analysis approach and why this was felt to be the most appropriate method.

Answer 3.9 We added two references at the following sentence *“In this qualitative study, we conducted semi-structured interviews with a thematic analysis (4, 5).” (*Page 3)

REFERENCES

1. Wilson JMG, Jungner G, World Health O. Principles and practice of screening for disease. Geneva: World Health Organization; 1968.
2. Wong KC, Nguyen TN, Trankle SA, Usherwood T, Quintans D, Marschner S, et al. Implementing a remote self-screening programme for atrial fibrillation using digital health technology among community-dwellers aged 75 years and older: a qualitative evaluation. BMJ Open. 2024;14(10):e088260.
3. Brik T, Lucassen WAM, Harskamp RE, Karregat EPM, Himmelreich JCL, Busschers WB, et al. Personalized approach using wearable technology for early detection of atrial fibrillation in high-risk primary care patients (PATCH-AF): Study protocol for a cluster randomized controlled trial. Am Heart J. 2022;254:172-82.
4. Braun V, and Clarke V. Using thematic analysis in psychology. Qualitative Research in Psychology. 2006;3(2):77-101.
5. Kiger ME, and Varpio L. Thematic analysis of qualitative data: AMEE Guide No. 131. Medical Teacher. 2020;42(8):846-54.
